# Synthesis and Photocatalytic Activity of Pt-Deposited TiO_2_ Nanotubes (TNT) for Rhodamine B Degradation

**DOI:** 10.3389/fchem.2022.922701

**Published:** 2022-05-31

**Authors:** Xiaojian Qiu, Zhenning Wan, Mengjie Pu, Xiuru Xu, Yuanyao Ye, Chunhua Hu

**Affiliations:** ^1^ School of Resources and Environment, Nanchang University, Nanchang, China; ^2^ College of Life and Environmental Science, Wenzhou University, Wenzhou, China; ^3^ School of Agricultural and Biological Technology, Wenzhou Vocational College of Science and Technology, Zhejiang, China; ^4^ School of Environmental Science and Engineering, Huazhong University of Science and Technology, Wuhan, China

**Keywords:** photocatalysis, TNT-Pt, morphology and structure, Rh B, superoxide radical anions

## Abstract

Dye wastewater has attracted more and more attention because of its high environmental risk. In this study, a novel TiO_2_ nanotube (TNT) catalyst was prepared and its morphology and structure were characterized. The synthetic catalyst was used to degrade Rhodamine B (RhB) under UV light and evaluated for the application performance. According to the characterization results and degradation properties, the optimum synthetic conditions were selected as 400°C calcination temperature and 10 wt% Pt deposition. As a result, the degradation efficacies were sequenced as TNT-400-Pt > TNT-500-Pt > TNT-400 > TNT-300-Pt. In addition, the effect of pH and initial concentration of RhB were explored, and their values were both increased with the decreased degradation efficacy. While the moderate volume of 11 mm of H_2_O_2_ addition owned better performance than that of 0, 6, and 15 mm. Scavengers such as tertbutanol (*t*-BuOH), disodium ethylenediaminetetraacetate (EDTA-Na_2_), and nitroblue tetrazolium (NBT) were added during the catalytic process and it proved that superoxide radical anions $\left(O_{2}^{–•}\right)$ , photogenerated hole (h^+^) and hydroxyl radical (OH•) were the main active species contributing for RhB removal. For the application, TNT-Pt could deal with almost 100% RhB, Orange G (OG), Methylene blue (MB), and Congo red (CR) within 70 min and still kept more than 50% RhB removal in the fifth recycling use. Therefore, TNT-Pt synthesized in this study is potential to be applied to the dye wastewater treatment.

## Introduction

With increasing technological and industrial development, a diverse set of pollutants have been discharged into water bodies, leading to the increasing concern about water contamination and environmental risks (Xu et al., 2021; Zeng et al., 2021). Colored dyes, represented by Rhodamine B (RhB), Orange G (OG), methylene blue (MB), and Congo red (CR) are widely used in the textile, printing, and plastic industries, which have high concentration levels in wastewater (Skjolding et al., 2021). Seriously, most dye pollutants cannot be easily degraded in water due to their complex composition, deep color, and chemical and physical stability (Sutar et al., 2022). Therefore, several techniques, such as adsorption, coagulation, biodegradation, and photocatalysis have been used in treating dyeing wastewater (Hao et al., 2021; Liu et al., 2021; Pu et al., 2017). Among these technologies, photocatalysis is increasingly regarded as a favorable option in recent years due to the advantages of its simple operation process, low energy consumption, and comparatively high degradation efficacy for pollutant removal (Xu et al., 2017a; Xu et al., 2017b).In addition, compared with photolysis, photocatalysis has the synergistic benefit of a specific catalyst combined with light irradiation (Xu et al., 2018; Xu et al., 2020a). Among many candidates of photocatalysts, TiO_2_ is the most widely studied material, currently the most likely photocatalyst for industrial-scale application in terms of high chemical stability, durability, high hydrophilicity, photoactivity efficiency, low toxicity, and low cost (Xu et al., 2020b; Hao et al., 2022).

While during photocatalysis by TiO_2_, the high recombination rates of photogenerated electron-hole pairs result in reduced photocatalytic efficiency (Perera et al., 2012). Accordingly, a series of strategies for the preparation of TiO_2_-based nanocomposites have been developed (Mi et al., 2021; Wang et al., 2021; Wu et al., 2021). TiO_2_-based nanotubes (TNTs) were first synthesized by electrochemical deposition in a porous aluminum oxide mold (Hoyer, 1996). Compared with commonly used TiO_2_ nanoparticles (NPs), TNTs exhibit unique photocatalytic properties including larger specific surface area (up to 478 m^2^/g) and larger pore volume (up to 1.25 cm^3^/g), comparatively strong ion-exchange capability; significant fast and long-distance electron-transport ability; and enhanced light absorption due to the high tube diameter ratio (Liu et al., 2014).

In addition, noble metals (e.g., Pt, Pd, and Ag) with TiO_2_ deposited can bend the valence band (VB) and conduction band (CB) as the difference in the Fermi level between metals and semiconductors (SC) to form a Schottky barrier (Christoforidis and Fornasiero, 2017). The work function (φ) increases with the greater Schottky barrier in the metal-SC heterojunction, causing a better charge separation effect, which is a key step in most photocatalytic processes. For example, TiO_2_ modified with Pt, Pd, and Ag has higher decomposition activity for the pollutants removal, with the pseudo-first-order kinetic rate constants of were 0.7267, 0.4369, and 0.1257 h^−1^, which were 12.5, 7.5, and 2.2 times higher than that of pure TiO_2_, respectively (Li et al., 2016). While among these noble metals, platinum (Pt) has a comparatively high work function (φ = 5.93 eV) with good performance as a TiO_2_co-catalyst (Fu et al., 2008; Chiarello et al., 2010). The reason has been explained that photogenerated electrons are used more efficiently in Pt atoms (Nguyen and Juang, 2019). In this study, TNT-Pt was prepared by hydrothermal synthesis, calcined at different temperatures (300, 400, 500°C), and different amounts of Pt loading (3, 5, 10, and 20 wt%). All catalysts were characterized for their morphology and structure and tested for the degradation performance of RhB in the photochemical reactor under UV irradiation. Meanwhile, the solution pH, initial concentration of the pollutants, and H_2_O_2_ addition affecting the degradation efficacy was discussed and the photocatalytic mechanism was explored by quenching experiments.

## Materials and Methods

### Preparation of TNT-Pt

Briefly, 1.2 g commercial TiO_2_ (AeroxideP25) and 75 ml NaOH aqueous solution (10 M) were placed in a 100 ml Teflon lined hydrothermal autoclave reactor and kept in an oven at 110°C for 12 h. Then, the sample was washed several times with deionized water and filtered. Afterward, the sample was ultrasonically treated with 0.1 M HCl aqueous solution for 15 min and filtered. After filtering, the sample was washed several times with deionized water. The obtained samples were kept in an oven at 80°C for 12 h. After being completely dried, the samples were collected and ground, which were identified as TiO_2_ nanotubes (TNT). All chemicals are of analytical grade. TNT was calcined at different temperatures in a tube furnace (Nabertherm P330) with the following temperature program: from 25°C to the calcination temperatures (T_calc_, °C) at a heating rate of 5°C/min, and keeping 3 h at T_calc_. The T_calc_ values were set at 300, 400, and 500°C, respectively. These three samples are labeled as TNT-300, TNT-400, and TNT-500.

Pt was photo-deposited on all the samples (P25, TNT, TNT-300, TNT-400, TNT-500). Firstly, 50 mg photocatalyst was added into 1.05 ml H_2_PtCl_6_ aqueous solution (10 g/L) to prepare 50 ml solutions and then mixed with 4 ml CH_3_OH. After irradiating under the 300 W mercury lamp for 3 h, the suspension was washed with deionized water and filtered to obtain the precipitate. After keeping in an oven at 80°C for 12 h, the sample was collected and determined to be TNT-Pt, TNT-300-Pt, TNT-400-Pt, and TNT-500-Pt, which were subsequently used in this study.

### Catalyst Characterization

The crystalline structure of the samples was determined by an XRD PANalytical Empyrean diffractometer, a Cu Kα radiation of 1.54 Å, scan step-size 0.0167°and a 2θ scan range of 10–90°. Absorption spectra of doped and undoped Pt samples were analyzed using a UV spectrometer (Shimadzu) scanning wavelengths from 200 to 800 nm. TEM and STEM-EDS analysis were performed by using Tecnai G2 and Titan FEI transmission electron microscopes, operating at 200 and 300 kV, respectively. The sample was prepared by suspending the powder in 2-propanol, ultrasounds treated, and finally dropping 5 μL of the suspension three consecutive times on a 400-mesh Cu grid provided by Tedpella, letting the solvent evaporate at room temperature. The specific surface area and pore volume of the derived nanotubes were determined by BET (Micromeritics, ASAP 2460/2020). Determination of Pt loading on TNT-Pt by ICP-MS (Agilent 7700s). Zeta potential values were determined using a laser particle size zeta potential analysis (Malvern Zetasizer Nano As). Zeta potential was measured three times at each pH value. The preparation method referred to the previous studies by some modifications (Xiong and Xu, 2016; Scandura et al., 2019).

### Photocatalytic Evaluation

The photodegradation of RhB in water was performed in a photochemical reaction instrument, which consisted of a 100 W mercury lamp with a wavelength of 365 nm, a condensation cup, and a magnetic stirrer inside a box. For degradation of RhB, the synthesized catalyst samples were added to the RhB solution with an initial concentration of 20 mg/L. Then, the suspensions were strongly stirred for 0.5 h in the dark to reach the adsorption equilibrium state. After that, the solution was exposed to UV irradiation for 70 min. During the photocatalytic process, 2 ml solution was sampled every 10 min (8 times in total) and filtered to remove the catalyst. The supernatant was analyzed to measure the concentration of RhB with a Hitachi UV-3010 UV-vis spectrometer. All experiments were conducted in triplicate.

## Results and Discussion

### Morphologies and Structures

**FIGURE 1:**
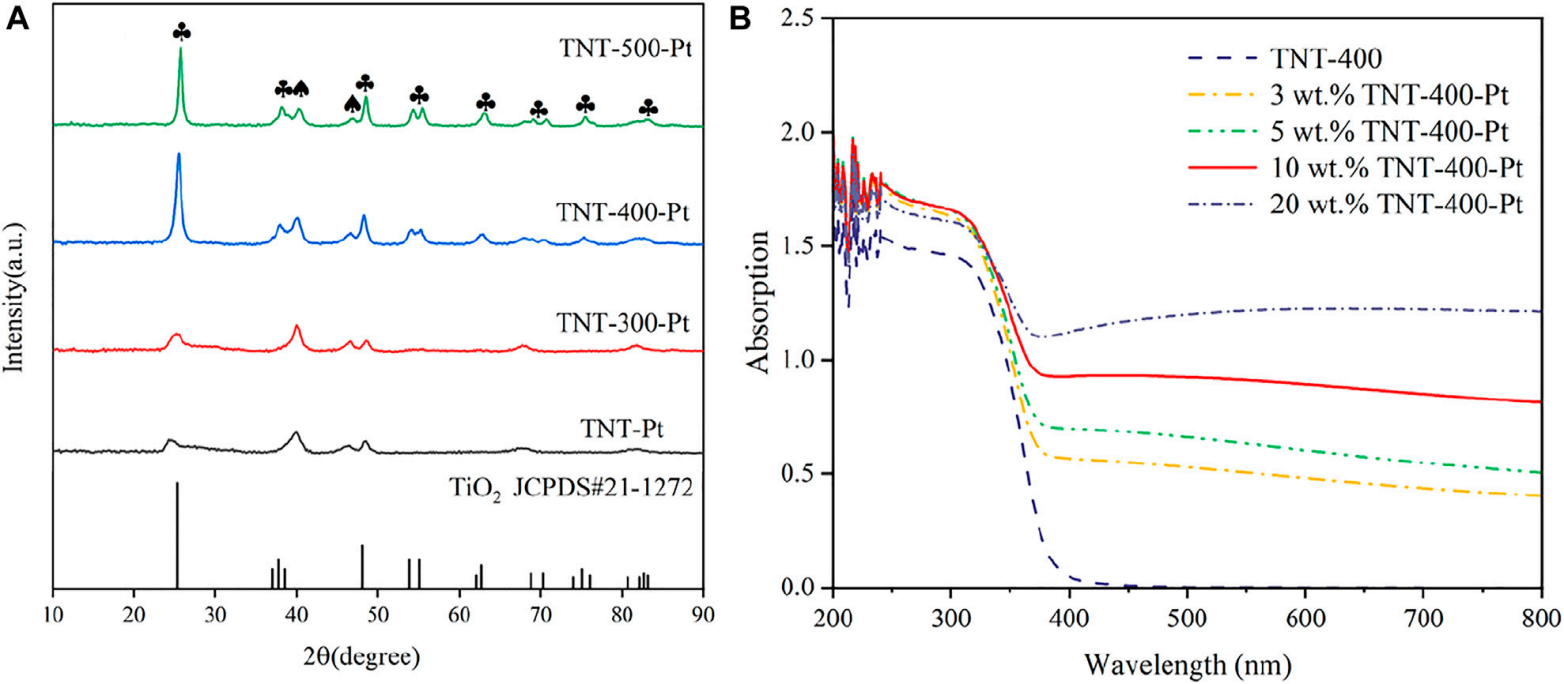
XRD patterns of different samples with most important planes indicated **(A)**, and UV absorption of samples with different Pt loading **(B)**, ♣: the typical diffraction peak of anatase, ♠: the diffraction peak of Pt oxide.

The evaluation of the phase and structure of the calcined TNT was observed through XRD patterns (Figure 1A). The crystallinity of these samples gets higher with the increase in calcination temperature (T_calc_). It can be seen that TNT-300 has only a small amount of anatase diffraction peaks (Supplementary Figure S1). The spectra of TNT-400 show characteristic diffraction peaks located at 25.281°, 37.8°, and 48.049°, corresponding to the (101), (004), and (200) reflection plane (JCPDS card 21-1,272) (Lazarte et al., 2018). H_2_Ti_3_O_7_ (202), brookite (200), and rutile (210) reflection plane appear in TNT-500 at 24.670°, 33.050°, and 44.699°. Thus, TNT-400 owns the highest amount and purest anatase type crystal phase than that TNT-300 and 500. After Pt was deposited on the surface of TNT, new diffraction peaks at 40.186° and 63.024°that derived from PtO_2_ (101) and PtO (222), respectively, appeared in the catalyst samples (JCPDS card 38-1355 and 47-1171). Moreover, the addition of Pt only passivates the diffraction peaks of anatase and does not affect the overall crystal form of the sample. In order to determine the photo absorbance properties, the UV absorption of TNT and Pt-TNT under different Pt loadings were analyzed by UV-Vis at wavelengths of 200–800 nm as shown in Figure 1B. The main light absorption wavelength of TNT-400 is in the ultraviolet range. However, with increasing Pt loading, the amount of visible light absorbed by the catalyst steadily increased, with only a slight increase in the amount of light absorption in the UV range, where 10 wt% loadings showed the best absorption of UV light in the UV rangeability. This illustrates the increased photosensitivity of Pt-modified TNTs in the visible and near-visible light wavelength range relative to pure TNTs.

**FIGURE 2:**
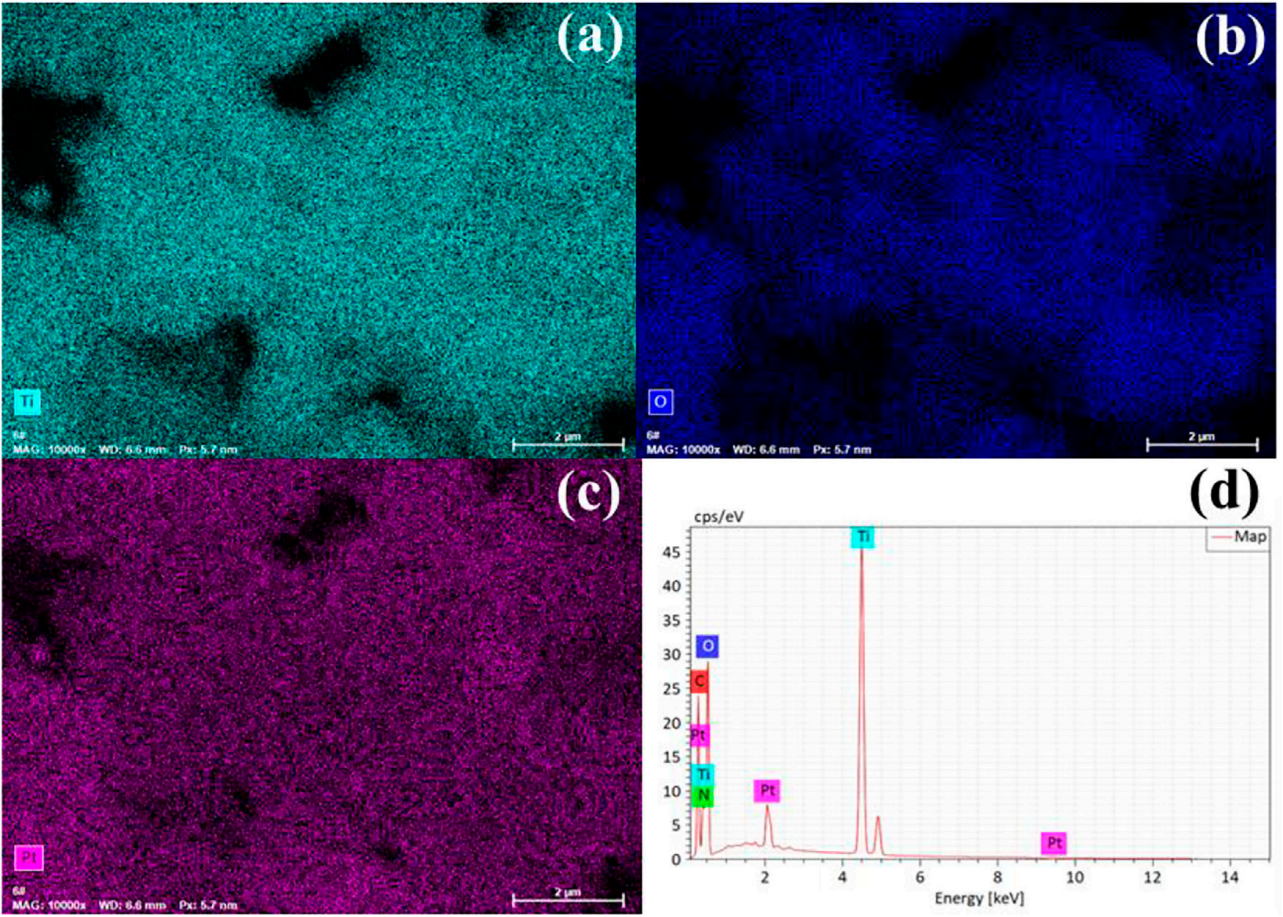
EDS mapping of elements Ti **(A)**, O **(B)**, Pt **(C)**, and elements composition **(D)** of TNT-400-Pt.

**FIGURE 3:**
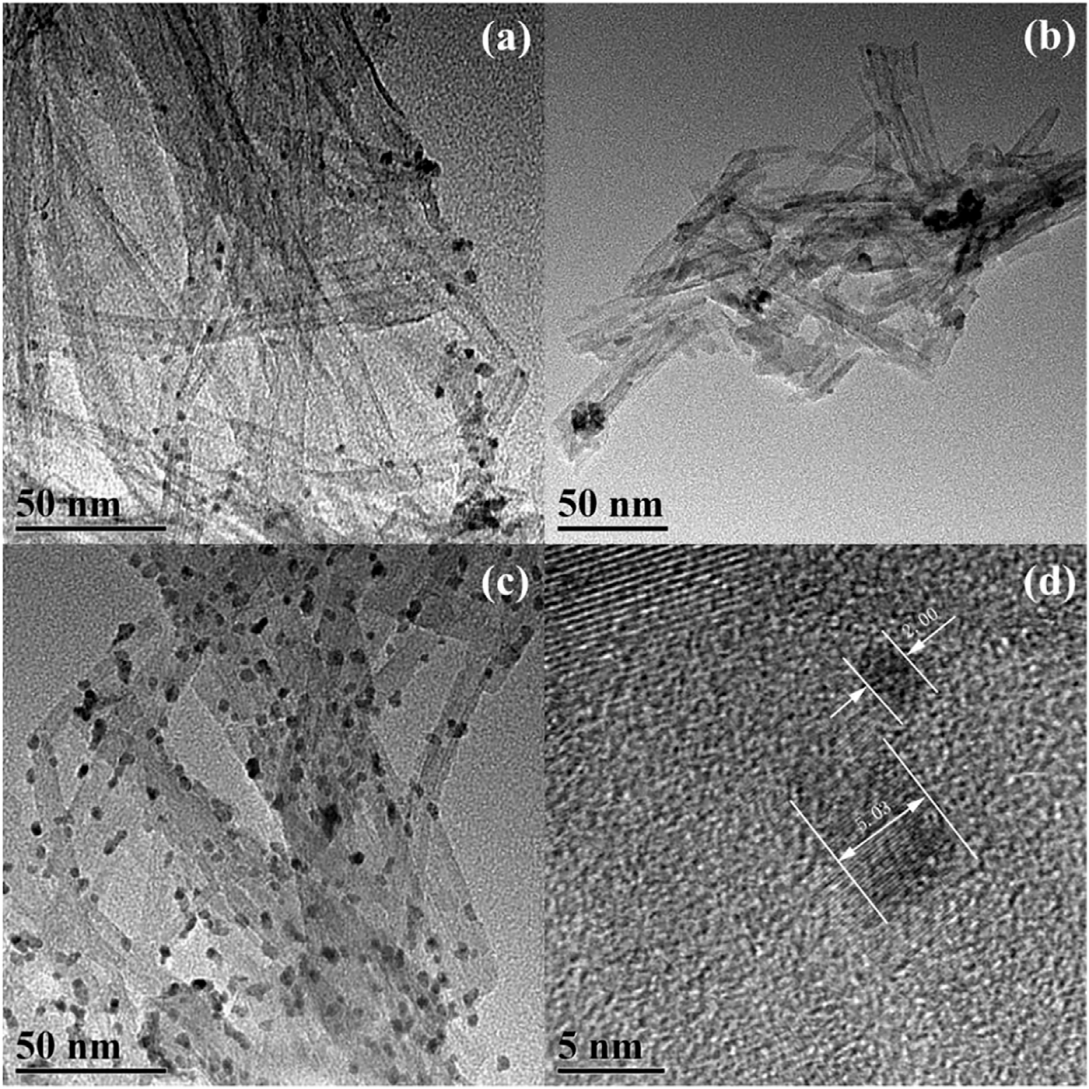
TEM images of TNT-300-Pt **(A)**, TNT-400-Pt **(B),** and TNT-500-Pt **(C)**, scale bar: 50 nm, Pt nanoparticle size **(D)**.

Furthermore, TNT-300, 400, and 500 were modified with Pt deposition, respectively. While no obvious change in the morphology of Pt-loaded samples was observed in SEM images (Supplementary Figure S2). EDS results verified the presence of Ti, O, and Pt elements (Figure 2), indicating the successful photo-deposition of Pt particles. BET results provided in Table 1, claim that the surface areas were followed the sequence as TNT-300-Pt = 286.2 m^2^/g > TNT-400 = 155.8 m^2^/g > TNT-400 = 148.2 m^2^/g > TNT-500-Pt = 81.5 m^2^/g proving maximum specific surface area of TNT-300 Pt. TEM was used to further analyze the morphology of the TNT-300, 400, and 500-Pt samples as shown in Figure 3. From the images, all the TNT tubes present a uniform distribution with an average diameter of 7–10 nm, with an opened tube orifice (Figures 3A–C), while the surface-adsorbed Pt nanoparticles exhibit a size of about 2–5 nm (Figure 3D), demonstrated again the emergence of new photocatalytic sites. A layered structure with an apparent edge was observed, illustrating the incomplete curling of part of the TNT tube. With the increase of calcination temperature from 300 to 500 °C, TNT tube curling degree increased and the loading content of Pt nanoparticles raised (Figures 3A–C).

**TABLE 1:** Surface areas and particle sizes of TNT-400 and TNT-Pt photocatalysts.

Catalyst	Surface areas (m^2^/g)	Pore volume (cm^3^/g)	Pore sizes (nm)
TNT-400	155.8403	1.0586	23.632
TNT-300-Pt	286.2142	1.005651	12.3963
TNT-400-Pt	148.194	0.646873	13.9094
TNT-500-Pt	81.4997	0.450061	17.1476

### Photocatalytic Behavior

**FIGURE 4:**
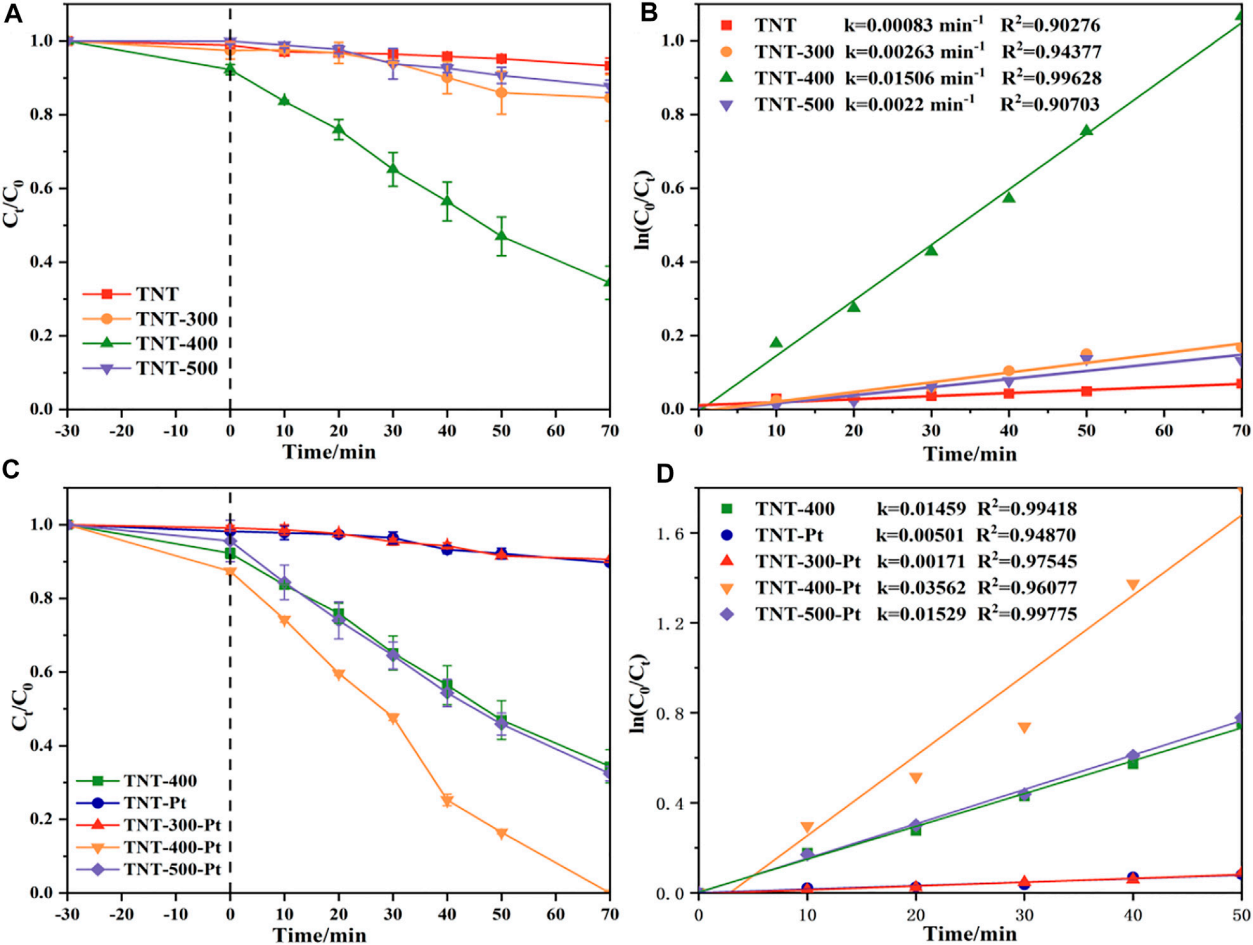
**(A)** Photocatalytic decomposition of RhB and **(B)** pseudo-first-order kinetic model by TNT, TNT-300, TNT-400, and TNT-500, **(C)** photocatalytic decomposition of RhB, and **(D)** pseudo-first-order kinetic model by TNT-Pt, TNT-300-Pt, TNT-400-Pt and TNT-500-Pt, pH = 6.83, initial RhB concentration = 20 mg/L.

The irradiation time versus the RhB concentration curves has been given in Figure 4. Obviously, TNT prepared under T_calc_ 400°C (TNT-400) exhibited higher photocatalytic ability for RhB removal than that of TNT-300, 500, and primitive material (TNT) as shown in Figures 4A,B. This could be explained that TNT-400 owns the highest amount of anatase type crystal phase according to the characterization results mentioned above. Commonly, the anatase type crystal phase contains more defects and vacancies, resulting in more oxygen vacancies to capture electrons, so it has higher activity (Li et al., 2021). Comparatively, TNT-300 and 500 own fewer amounts of anatase type crystal phase, especially TNT-500 with part of rutile type crystal phase, which has almost no photocatalytic activity (Phuong and Yoo, 2020). Thus, TNT-300 and 500 had poor photocatalytic capacity for RhB Removal. While after the Pt loading, the degradation performance was highly promoted as RhB was completely bleached by TNT-400-Pt within 70 min (Figure 4C). The kinetics of the photodegradation of RhB fitted well to the pseudo-first-order model (R^2^ > 0.90) based on Eq. 1 (Mansurov et al., 2022):

$$\text{In}\left({\text{C}}_{0}/{\text{C}}_{\text{t}}\right)=\text{k}\text{t}$$ (1)

where k is the rate constant, C_0_ and C_t_ are the concentration of RhB in solution at irradiation time 0 and t (min^−1^), respectively. As shown in Figure 4D, the rate constant (k) was ranked as k_TNT-400-Pt_ = 0.035 min^−1^ > k_TNT-400_ = 0.015 min^−1^. As Pt loading on TNT was favorable for O_2_ adsorption and the superoxide radical $\left(O_{2}^{–•}\right)$ formation, which plays the key role for RhB degradation. Similarly, TNT-400-Pt owned better catalytic ability than TNT-300, 400 and original TNT-Pt, with the sequence of k value as k_TNT-400-Pt_ = 0.035 min^−1^ > k_TNT-500-Pt_ = 0.015 min^−1^ > k_TNT-300_ = 0.002 min^−1^ ≈ k_TNT-Pt_ = 0.002 min^−1^, which further proved the high activity of TNT-400-Pt. This might be attributed to the various Pt contents deposited in TNT. Under different T_calc_. the Pt contents deposited in TNT-300, 400, 500 detected by ICP-MS were 7.3 wt%, 7.5 wt%, and 6.8 wt%, thus TNT-400-Pt owned the highest amount of Pt deposited in the catalyst. Furthermore, TNT-400 owns the highest amount of anatase type crystal phase, leading to the stronger photocatalytic ability for pollutant removal.

**FIGURE 5:**
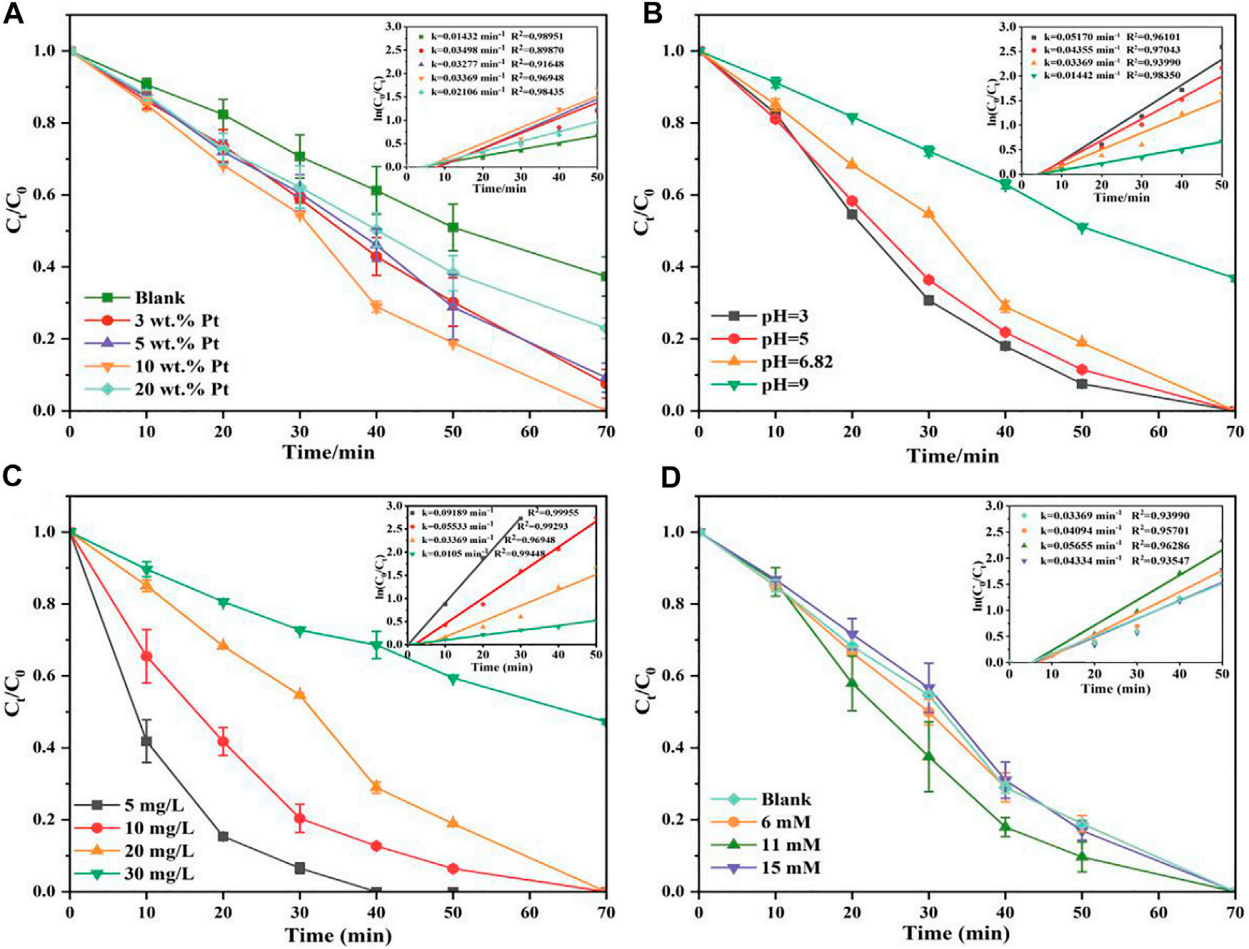
Change Pt load on TNT-400 **(A),** effects of pH_ini_ **(B)** on RhB photodegradation by TNT-400-Pt (10), the effect of initial concentration of RhB on the photocatalytic degradation of TNT-400-Pt (10) **(C)**, effects of H_2_O_2_ **(D)** on RhB photodegradation by TNT-400-Pt.

In this case, the effects of different experimental factors were investigated on photocatalysis by TNT-400-Pt. First, the loading amount of Pt during the synthesis process is essential for the catalytic performance as shown in Figure 5A. As a result, only a proper amount of Pt 10 wt% loading has a positive effect on RhB removal. A higher or lower amount of Pt (20 wt% or 5 wt% and 3 wt%) loading had the reduced degradation efficacies, which were all better than that of pure TNT. This could be explained that Pt deposition could provide active species for the pollutant oxidation, while the excess loading may cover active sites on the TiO_2_ surface, thereby reducing photodegradation efficiency, which was also discussed by previous literature (Shawky et al., 2020). In addition, the UV-vis results (Figure 1B) exhibit that 20 wt% TNT-Pt had higher absorption values than that of others, which also could be the reason for its higher degradation performance.

Moreover, the photodegradation of RhB by TNT-400-Pt (10 wt%) was evaluated at various initial solution pHs of 3, 5, 7, and 9 as shown in Figure 5B. The degradation efficacy is higher under acid conditions than that under neutral and alkali conditions, which is probably due to fact that the charge of TNT-400-Pt at the pH of 6.1 is zero as shown in Supplementary Figure S3 in the appendix. This suggests that the TNT surface was positively charged at pH < 6.1, while negatively charged at pH > 6.1. At low pH, H^+^ adsorbed on the catalyst surface has a large proton exchange capacity, which could react with the photogenerated electrons to form hydrogen radical (H•). Meanwhile, a lower pH solution has electronegative centers, leading to the promoted adsorption on the surface of TiO_2_, which also increase the degradation rate under acid condition. A similar explanation has also been mentioned in the previous literature (Mohanty et al., 2020).

Figure 5C exhibits the effect of initial concentration in the range of 5–25 mg L^−1^ on the catalytic performance. The results show that the degradation efficacy was highest at the RhB concentration of 5 mg L^−1^. While it decreased with the concentration increasing as the rate constant (k) was ranked from highest to lowest as k_5 mg/L_ = 0.092 min^−1^ > k_10 mg/L_ = 0.055 min^−1^ > k_20 mg/L_ = 0.034 min^−1^ > k_30 mg/L_ = 0.011 min^−1^, which could be explained as the active radicals generated on the catalyst surface were reduced due to the occupation of pollutant molecules in the active sites. Furthermore, H_2_O_2_ reported as an electron acceptor also plays role in the RhB removal. Figure 5D reveals that H_2_O_2_ in the RhB solution enhanced the degradation efficacy, especially 11 mm addition with the promoted rate constant of 0.057 min^−1^, higher than that of 7 mm and no H_2_O_2_ addition. The reason could be attributed to the generation of hydroxyl radical (OH•) reacting from the reaction of H_2_O_2_ with electron (e^–^) as the Eq. 2 (Wang J.-F. et al., 2022).

$$e^{-}+H_{2}O_{2}\to OH^{-}+\mathrm{OH}•$$ (2)

While a limiting value for the degradation rate occurred when the H_2_O_2_ addition achieved 15 mM due to the scavenging reaction as shown in Eq. 3.

$$H_{2}O_{2}+\mathrm{OH}•\to H_{2}O+HO_{2}•$$ (3)

### Photocatalytic Mechanism

During the photocatalytic process, the main active species include photogenerated holes (h^+^) and electrons (e^–^), hydroxyl radicals (OH•), and superoxide radical anions $\left(O_{2}^{–•}\right)$ that could be produced based on the following Eq. 4–6.

$$\text{TNT}-\text{Pt}+\text{hv}\to \text{TNT}-\text{Pt}\left({\text{h}}^{+}+{\text{e}}^{-}\right)$$ (4)

$$h^{+}+{\text{H}}_{2}\text{O}\to \text{OH}•+{\text{H}}^{+}$$ (5)

$${\text{e}}^{-}+{\text{O}}_{2}\to {\text{O}}_{2}^{-•}$$ (6)

**FIGURE 6:**
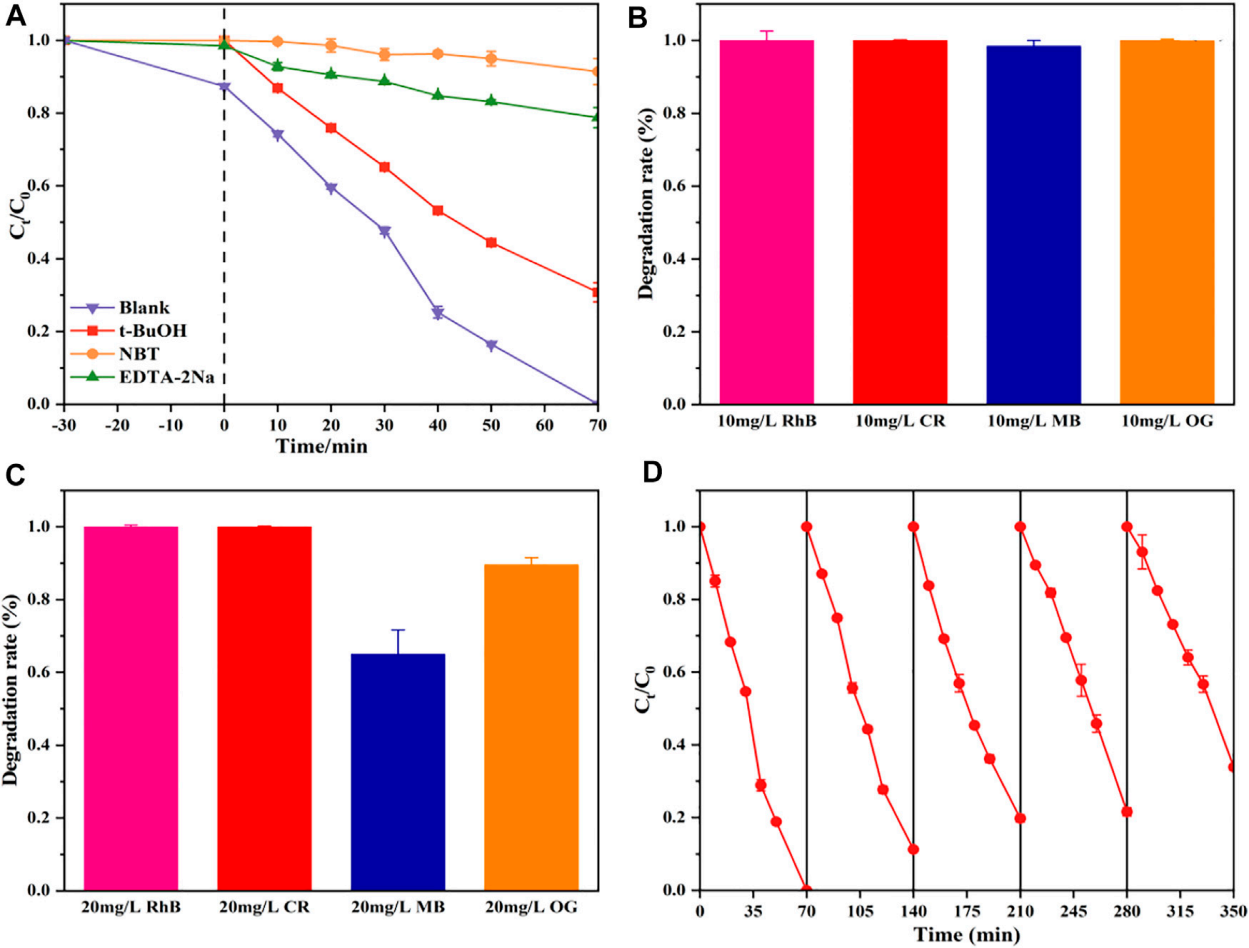
Photocatalytic activities of the TNT-400-Pt sample for RhB degradation with disparate scavengers **(A)**, photocatalytic degradation of different dyes within 70 min by TNT-400-Pt at an initial concentration of 10 mg/L **(B),** and 20 mg/L **(C)**, five cycles of degradation of TNT-400-Pt **(D)**.

**FIGURE 7:**
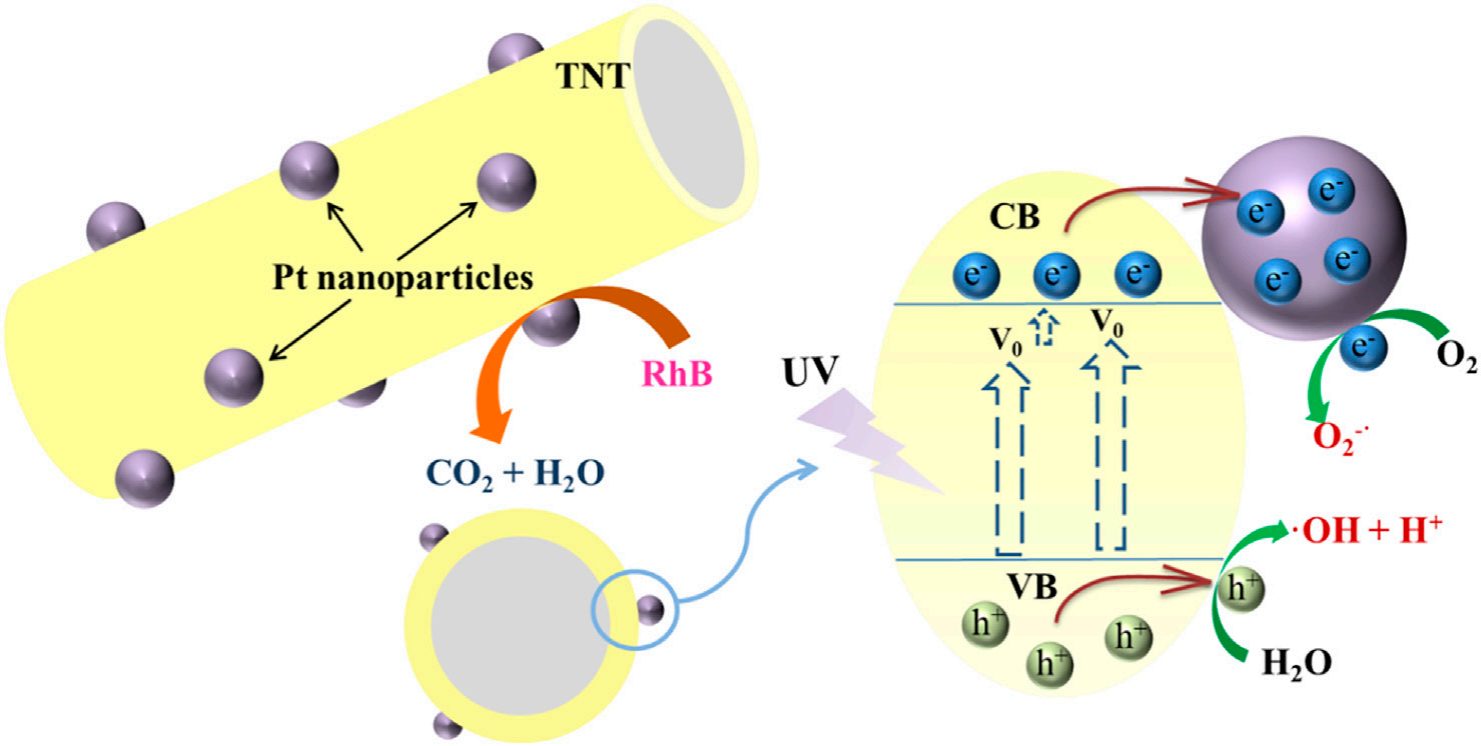
The possible photocatalytic mechanism of TNT-PT for RhB removal.

To confirm the significance of these active species, tertbutanol (*t*-BuOH), disodium ethylenediaminetetraacetate (EDTA-Na_2_), and nitroblue tetrazolium (NBT) as the scavengers of OH•, h^+^, and $O_{2}^{–•}$ , respectively were added during the photocatalytic process. As shown in Figure 6A, it could be easily observed that the degradation efficacy was poorest with NBT addition, followed by EDAT-Na_2_ and *t*-BuOH addition, compared with the performance by TNT-Pt without scavengers. This proves that $O_{2}^{–•}$ plays an essential role in the RhB degradation, then was a photogenerated hole (h^+^) and OH• contributed to the pollutant removal. Accordingly, the possible photocatalytic mechanism could be speculated as shown in Figure 7. Under UV irradiation, the RhB molecules were activated as short-lived active transient, adsorbed over Pt metal sites. Meanwhile, Pt loading induced the $O_{2}^{–•}$ generation of pollutant oxidation, and nano-structure holes of TNT could provide unique space and electronic environments for Pt active sites, which was easier to inhibit the recombination of photogenerated electron and hole pairs, leading to the prior photocatalytic ability dealing with RhB. This was consistent with the literature (Ding et al., 2022; Wang X. et al., 2022). Therefore, the catalytic activity for RhB was significantly improved by TNT with Pt deposition.

### Application to Other Dye Pollutants and Recycling

TNT-Pt-400 (10wt%) was also used for other dye pollutants such as CR, MB, and MO as shown in Figures 6B,C. As a result, at initial concentrations of 10 mg/L, almost all RhB, CR, MB, and OG could be removed within 70 min by TNT-Pt. While at the concentration of 20 mg/L, 100% RhB and CR, 65% MB and 90% OG could be degraded within 70 min. Thus, the obtained catalysts of TNT-Pt own a comparatively strong capacity for dye pollutants removal. In addition, the reuse capacity of the synthesized materials (TNT-Pt-400, 10 wt%) was evaluated by five cycling usages as shown in Figure 6D. Obviously, the performance stained well as almost 100% RhB removal in the first three cycles, 80% removal remained in the third time of cycling, and more than 60% removal was achieved in the fifth time, indicating that the synthesized catalysts were reusable and exhibited high potential on the applications of real wastewater treatment.

## Conclusion and Future Perspective

In summary, TNT-Pt was synthesized successfully and exhibited well-characterized morphology and structure. During the synthetic process, 400°C calcination temperature and 10 wt% Pt deposition was determined to be the preferable condition to form a better crystal morphology based on the characterization results. In the photodegradation experiments, the rate constant (k) was ranked as k_TNT-400-Pt_ = 0.045 min^−1^ > k_TNT-400_ = 0.014 min^−1^. In addition, acid solution (pH 3) and lower initial concentration of RhB (5 mg/L) both increased the degradation process, while a moderate volume of 11 mm H_2_O_2_ addition had the promoted degradation performance. Furthermore, in the quenching experiment, NBT had the most significant inhibition effect on the photocatalytic efficacy than other scavengers, suggesting the dominant active species $O_{2}^{–•}$ . Besides, the synthesized TNT-Pt could remove almost CR, MB, and OG as well as RhB, and its catalytic capacity stained well in five recycling usages.

So far, large datasets have existed on the synthesis of photocatalyst materials and their degradation ability for particular pollutant removal (Xu et al., 2020a). Nevertheless, information on the controlling factors of the photocatalysis process and the immobilization and recycling use of catalysts are limited. Thus, future research should focus on the follows:

◆ Assessing dissolved oxygen (DO) and dissolved organic matters (DOM) effect on the photocatalytic performance of the as-synthesized TNT-Pt;
◆ Exploring the immobilization of the synthesized catalysts when dealing with real dye wastewater;
◆ Utilizing the electron paramagnetic resonance (EPR) to detect the active radicals directly for the further investigation of the reaction mechanism;
◆ Synthesizing more functionalized yet low-cost catalyst polymers decomposing the dye water with high efficacy.

## Data Availability

The raw data supporting the conclusions of this article will be made available by the authors, without undue reservation.
