# Supplementary material for: Synthesis and Photocatalytic Activity of Pt-Deposited TiO2 Nanotubes (TNT) for Rhodamine B Degradation
Source: Front Chem. 2022 May 31;10:922701. doi: 10.3389/fchem.2022.922701 (PMC9194477; doi:10.3389/fchem.2022.922701)
Supplement: Supplementary file 1 [file Table1.DOCX]

Supplementary material for

**Synthesis and Photocatalytic Activity of Pt-deposited TiO_2_ Nanotubes (TNT) for Rhodamine B Degradation**

***Xiaojian Qiu^1^, Zhenning Wan^2^, Mengjie Pu^2^, Xiuru Xu^3*^, Yuanyao Ye^4^, Chunhua Hu^1*^***

*^1^ School of Resources & Environment, Nanchang University, Nanchang, Jiangxi, 330031, China*

*^2^ College of Life and Environmental Science, Wenzhou University, Wenzhou 325035, China*

*^3^ School of Agricultural and Biological Technology, Wenzhou Vocational College of Science & Technology, Zhejiang 325006, China*

*^4^ School of Environmental Science and Engineering, Huazhong University of Science and Technology, Wuhan 430074, China*

*Corresponding author at ** *Xiuru Xu,* *School of Agricultural and Biological Technology, Wenzhou Vocational College of Science & Technology, Zhejiang 325006, China. E-mail address: [wkyxxr@163.com](mailto:wkyxxr@163.com); Chunhua Hu, School of Resources & Environment, Nanchang University, Nanchang, Jiangxi, 330031, China. E-mail address:* [*chhu@ncu.edu.cn*](mailto:chhu@ncu.edu.cn)*.*


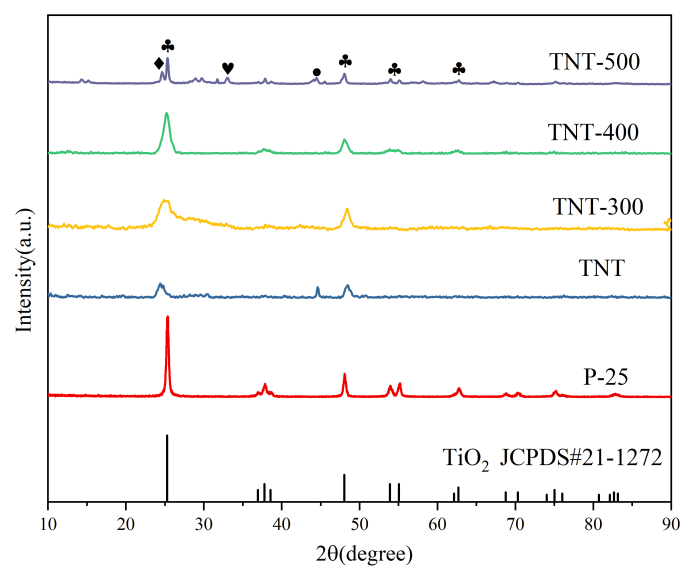


**Figure S1** XRD patterns of different samples with most important planes indicated, ♦ represents the diffraction peak of H_2_Ti_3_O_7_ , ♣ : the typical diffraction peak of anatase, ♥ represents the diffraction peak of brookite, ● : the diffraction peak of rutile.


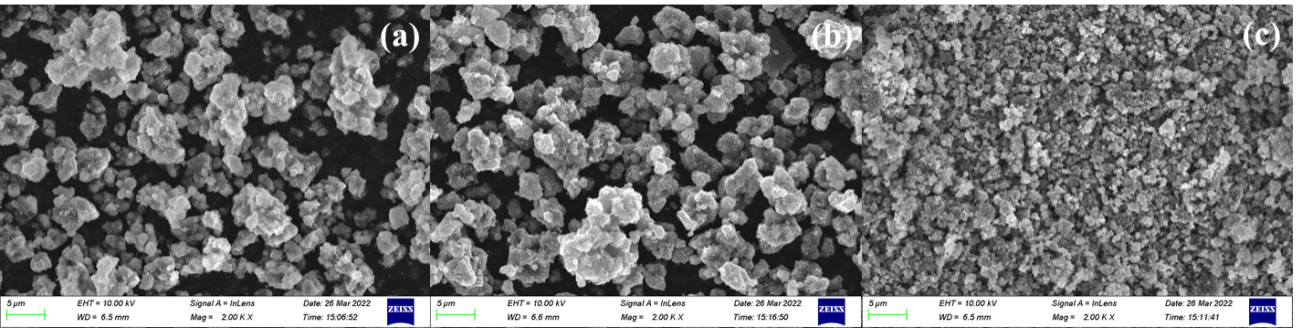


**Figure S2** Under the condition of different calcination temperature of TNT-Pt SEM images.


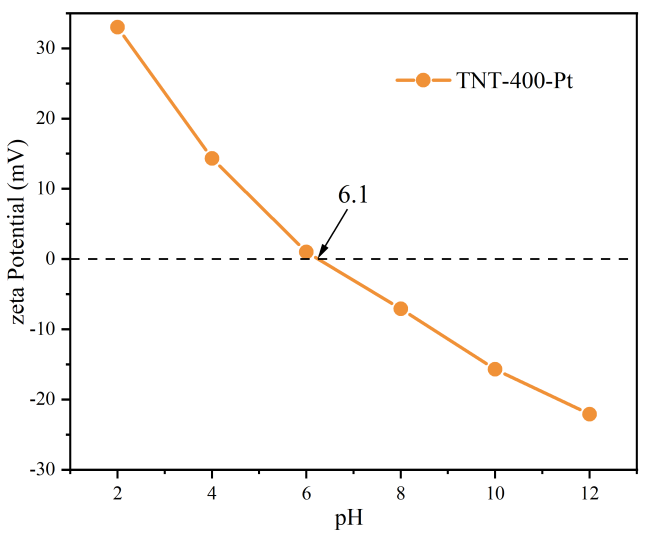


**Figure S3** The effect of pH on the zeta potential of TNT-400-Pt.
